# Unveiling nonessential gene deletions that confer significant morphological phenotypes beyond natural yeast strains

**DOI:** 10.1186/1471-2164-15-932

**Published:** 2014-10-25

**Authors:** Ming Yang, Shinsuke Ohnuki, Yoshikazu Ohya

**Affiliations:** Department of Integrated Biosciences, Graduate School of Frontier Sciences, University of Tokyo, Bldg. FSB-101, 5-1-5 Kashiwanoha, 277-8562 Kashiwa, Chiba Prefecture, Japan

**Keywords:** Yeast, *Saccharomyces cerevisiae*, Cell morphology, Phenotypic diversity, Gene function, Species conservation

## Abstract

**Background:**

Phenotypes are variable within species, with high phenotypic variation in the fitness and cell morphology of natural yeast strains due to genetic variation. A gene deletion collection of yeast laboratory strains also contains phenotypic variations, demonstrating the involvement of each gene and its specific function. However, to date, no study has compared the phenotypic variations between natural strains and gene deletion mutants in yeast.

**Results:**

The morphological variance was compared between 110 most distinct gene deletion strains and 36 typical natural yeast strains using a generalized linear model. The gene deletion strains had higher morphological variance than the natural strains. Thirty-six gene deletion mutants conferred significant morphological changes beyond that of the natural strains, revealing the importance of the genes with high genetic interaction and specific cellular functions for species conservation.

**Conclusion:**

Based on the morphological analysis, we discovered gene deletion mutants whose morphologies were not seen in nature. Our multivariate approach to the morphological diversity provided a new insight into the evolution and species conservation of yeast.

**Electronic supplementary material:**

The online version of this article (doi:10.1186/1471-2164-15-932) contains supplementary material, which is available to authorized users.

## Background

Evolution has produced remarkably complex and diverse living organisms with different morphological phenotypes observed in shape, size, and other traits. This morphological variation is important for their survival during environmental disruption, and many biologists aim to clarify how organisms evolved their phenotypic variation throughout their long evolutionary history.

The budding yeast *Saccharomyces cerevisiae* is a leading model organism used in genetics and systems biology because it has cellular processes in common with many eukaryotic cells. After the whole genome of the laboratory yeast strain was sequenced [1], most yeast genes were functionally annotated, providing insights into the relationship between genotype and phenotype. High genetic variance was found in different yeast subgroups based on analyses of yeast strains isolated from different ecological niches [2, 3]. Natural yeast strains also exhibited high phenotypic variation based on the analysis of yeasts cultured under various environmental conditions [4]. These results described the relationship between phenotype and genetic background, which provided insights into the origins of natural phenotypic variation. However, the relationship between the phenotype and genotype in natural strains remains unclear, as how an individual gene influences the phenotypic variation within the species is unknown. Furthermore, most previous research was restricted to fitness [4] and gene expression [5] phenotypes.

**Figure 1:**
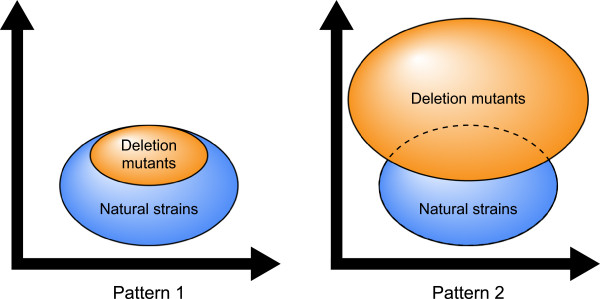
**Hypothetical model to compare phenotypic variations.** The set of gene deletion mutants (orange) and natural strains (blue) are compared in orthogonal phenotypic space. (Pattern 1) Variation of the natural strains is larger. (Pattern 2) Variation of the set of gene deletion mutants is larger.

Yeast cell morphology reflects various cellular events, such as progression through the cell cycle, establishment of cell polarity, and regulation of cell size. Recent techniques have allowed quantitative analysis of the relationships between different morphological traits and gene deletions. A study on 4718 haploid nonessential gene deletion strains revealed that approximately half showed abnormal morphological traits compared to the parental strain [6], and approximately 500 of these genes were thought to be phenotypic capacitors required for the maintenance of phenotypic robustness [7]. A recent study showed that the proportion of genes affecting a trait varies from <1% to >30%, averaging 6% [8]. The natural strains exhibited their own morphological traits [9]. However, how the natural strains confer such phenotypic variation remains unclear. One of the ways to resolve this is to compare morphological variations in natural strains and those generated by deleting individual genes (Figure 1). If the variations generated by the set of individual gene deletion mutants are smaller than those of the natural variation (Figure 1, pattern 1), this suggests that the most distinct morphologies among the natural strains are due to their genetic complexity. If the variations of the set of gene deletion mutants are larger (Figure 1, pattern 2), then that deleted gene beyond the natural strains is possibly functional in the natural strains and important for the maintenance of natural yeast morphology.

In this study, a high-dimensional image-processing program CalMorph was used on digital microscopic images to ascertain cell shape, actin, and nuclear DNA morphology. We analyzed the morphological variations with a generalized linear model (GLM), an extension of the normal linear model [10], by incorporating various probability distribution models. The statistical models were set to assess the effects of a homozygous gene deletion of yeast diploid on cell morphology.

## Results

### Mosaic segregants are more variable than their pure parental strains

To evaluate the morphological diversity in yeast, we focused on two aspects: the standard deviation in each parameter and the population expansion in the orthogonal phenotypic space that was degenerated in dimension from high-dimensional morphological traits. The diverse population could be expanded in orthogonal phenotypic space.

**Figure 2:**
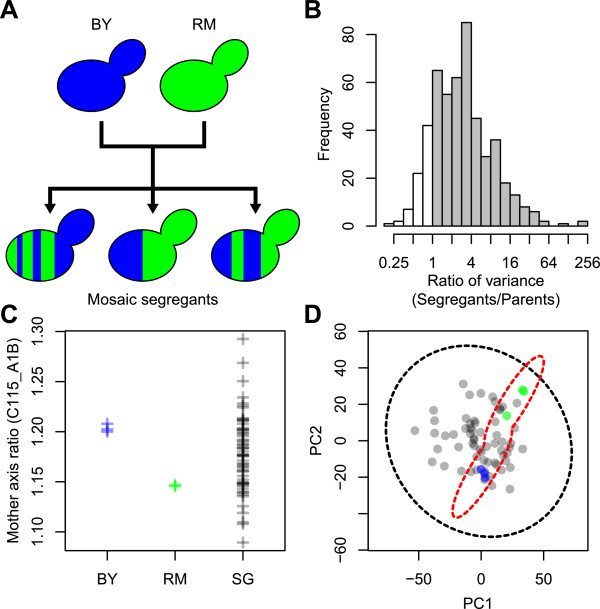
**Distribution of morphological phenotypes of BY, RM, and segregants. (A)** Schematic illustration of BY, RM, and segregants. Blue and green cells indicate the BY and RM lineages, respectively. Blue and green striped cells indicate segregants having a mosaic genotype by crossing BY and RM. **(B)** Distribution of the ratio of variance of the segregants to variances of BY and RM. Gray and white boxes indicate parameters with a larger and a smaller variance, respectively, of segregants than the mean square due to BY and RM. **(C)** Distribution of the mother axis ratio (C115_A1B). Each cross represents the estimated value from triplicate GLM values (see Methods). SG indicates segregants. **(D)** Equiprobability density ellipse of BY, RM, and segregants. Blue, green, and gray circles indicate BY, RM, and segregants, respectively. The red dashed ellipse denotes the Gaussian mixture of the multivariate normal distribution between BY and RM. The black dashed ellipse represents the multivariate normal distribution of segregants.

To confirm the validity of our procedure, we compared mosaic segregants with their pure parental strains, because it is well known that genetic mosaicism results in phenotypic variance [11]. Two pure parental strains belonging to the laboratory strain (BY) and the wine strain (RM) and their mosaic segregants (Figure 2A) were compared with the 501 morphological traits of the published data [12]. We analyzed the 501 morphological traits, including cell shape, actin, and nuclear DNA morphology, as previously described [6]. The morphological value of each trait was normalized using the GLM (see Methods). We found that parameters with a broader distribution were more frequently observed in the mosaic strains (Figure 2B). The variance in the mosaic segregants was higher than that in the parental strains for 427 of 501 traits. We also confirmed the diversity of the mosaic segregants in a representative trait, termed the “mother axis ratio” (C115_A1B; Figure 2C).

To ascertain the degree of global morphological variance, we performed principal components analysis (PCA), a statistical procedure that uses an orthogonal transformation. An advantage of comparing in the degenerated orthogonal space is that one can exclude bias caused by the correlation between the morphological parameters. We found that the morphological distribution of the mosaic segregants was broader than that of the parental strains in the principal component (PC)1 and PC2 spaces (Figure 2D). The mosaic segregants also showed a broader distribution in the spaces between any other pairs from PC1 to PC4 (Additional file 1: Figure S1). Thus, our analyses confirmed that the mosaic strains contained a higher morphological variance than that of the parental strains and validated our procedure.

### Homozygous gene deletion strains have higher morphological variance than natural strains

**Figure 3:**
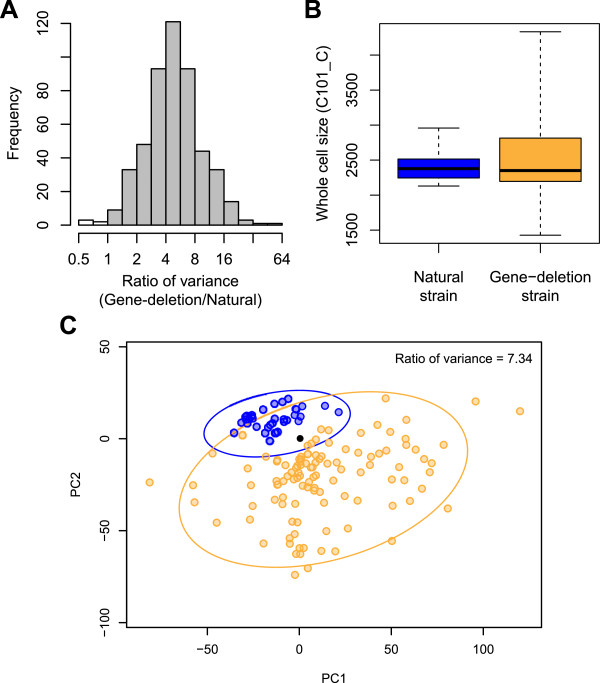
**Distribution of morphological phenotypes of the natural strains and the gene deletion strains. (A)** Distribution of the ratio of variance of the gene deletion strains to the natural strains. Gray and white boxes indicate the parameters with a larger and a smaller variance, respectively, of gene deletion strains than those of the natural strains. **(B)** Boxplot of the whole cell size (C101_C). **(C)** Equiprobability density ellipse of the natural strains and the gene deletion strains. The blue, orange, and black circles indicate the natural strains, the gene deletion strains, and BY4743, respectively. The blue and orange ellipses show the multivariate normal distribution of the natural strains and the gene deletion strains, respectively. Ratio of variance was calculated by gene-deletion/natural.

To compare the phenotypic variation between natural strains and gene deletion mutants, an analysis was performed of the morphological data of 36 typical natural yeast strains previously published [9] and the most distinct 110 gene deletion mutants. The most distinct homozygous diploid strains were selected based on the morphological data of the haploid strains, using global Mahalanobis distance, a unitless and scale-invariant measure of the distance and an abnormal distribution in the PC1 − PC20 phenotypic space (Additional file 2: Figure S2, Additional file 3: Table S1; see Methods). We confirmed the distinct morphological phenotypes of 110 gene deletion mutants in diploid (Additional file 2: Figure S2D). We found that the variance of the set of the gene deletion mutants was higher than that of the natural strains for 493 of 498 traits (99%; Figure 3A), while the most distinct 110 gene deletion mutants were more diverse in the “whole cell size” (C101_C) representative trait (Figure 3B). Analysis of the global variance also revealed more distinct gene deletion strains than found in the natural strains. The morphological distribution of the gene deletion strains was 7.34-fold broader than that of the natural strains in the first two PC spaces (Figure 3C). Diversity of the gene deletion strains was also observed in the spaces between any other PC1 − PC4 pairs (Additional file 4: Figure S3). Based on these results, we concluded that the set of the gene deletion strains had a higher morphological variance than the natural strains.

### Identification of mutants with a higher number of morphological changes than natural strains

**Figure 4:**
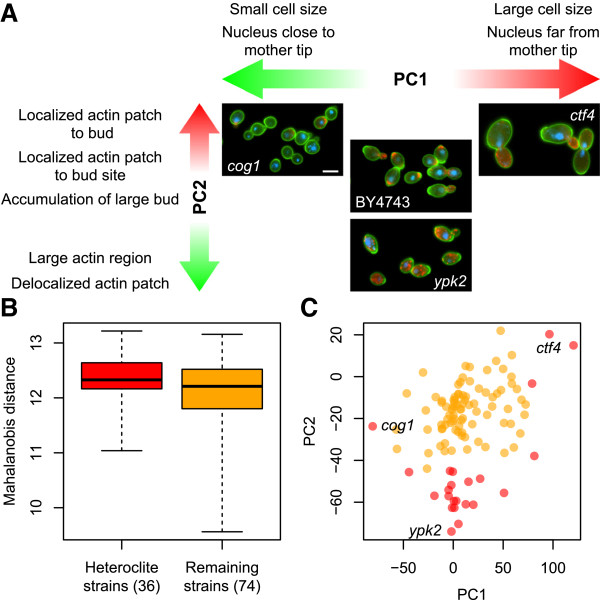
**Representative cell morphology and phenotypic distribution of the selected deletion mutants. (A)** Representative morphological features of each PC and images of yeast cells. PCs were characterized by the PC loadings (Additional file 8: Figure S6). Red and green arrows indicate an increase and decrease in the PC scores, respectively. Scale bar indicates 5 μm. **(B)** Boxplot of Mahalanobis distance. Mahalanobis distance from a center of distribution of BY4743 was calculated from the Z-scores (see Methods). **(C)** Distribution of PC scores of PC1 and PC2. Red and orange circles indicate the 36 detected strains and the 74 undetected strains, respectively. Images of the strains indicated in the plot were exemplified in **(A)**.

Given that the set of the gene deletion strains were more diverse, some of the deletion mutants were expected to have an abnormal morphology not seen in the natural yeast strains. To identify the deletion mutants with significant morphological changes beyond those of the natural strains, we performed a one-sample test with a normal distribution based on the PC scores in PC1 − PC4. The contribution ratio of the first four PCs reached 60% (Additional file 5: Figure S4). Thus, we detected the 36 heteroclite mutants (*P* <0.05, Bonferroni correction; Additional file 6: Table S2) with the first four PCs, expecting that the gene functions disrupted in these 36 mutants are important in nature. We found that the number of mutants detected in each PC was not the same, with 5, 18, and 17 mutants in PC1, PC2, and PC4, respectively. No mutants were detected in PC3. The mutants detected in each PC seldom overlapped. Only four overlapping mutants were detected in the 36 heteroclite mutants (Additional file 7: Figure S5A). PC2 and PC4 had a higher ability to detect the heteroclite mutants; of the 36 mutants, 32 (89%) were detected by PC2 or PC4 (Additional file 7: Figure S5B). These results suggested that the deletion mutants detected by PC2 and PC4 caused more diverse morphological changes than the natural strains. To know the morphological features that vary among strains, the PC loadings of PC1, PC2 and PC4 were analyzed (loadings >0.6, *P* <1.95 × 10^−3^ after Bonferroni correction; Additional file 8: Figure S6). Representative parameters were then extracted by a PCA with null-distributed data as described by Ohnuki et al. [13, 14]. The PCA analyses indicated that PC1 contributed most to the following traits: the “average cell size of budded cells” (C101_A1B) and “distance between nucleus and mother tip” (D103_C; Figure 4A). Likewise, PC2 contributed to the following traits: “ratio of the cells with actin localization in bud” (A112), “size of actin region in bud” (A7-1_A1B), and “ratio of large bud” (C122; Figure 4A). PC4 contributed to the traits, “Noise of long axis length in mother” (CCV103_A1B) and “Noise of mother cell size” (CCV11-1_C) as shown in Additional file 8: Figure S6. To ascertain whether sufficient deletion mutants had been analyzed, the next most distinct 20 gene deletion strains were sampled (Additional file 3: Table S1) and the one-sample test was repeated. No deletion strains were detected in the first four PCs, suggesting that our analysis of the morphologically variable 110 strains was robust.

### Properties of the 36 heteroclite mutants

Although the one sample test using the natural strains identified 36 heteroclite mutants out of 110 mutants, the average Mahalanobis distance from the parental strains was not significantly different (Mann–Whitney *U*-test, *P* <0.05; Figure 4B), suggesting that the difference between these groups was not due to the extent of morphological difference but rather to different morphological profiles. Because each PC represented different cell morphological features, we looked for differences in the PCs. Then we found that these 36 genes were distributed differently from those in the PC1 and PC2 spaces (red dots in Figure 4C). Some of the deletion mutants with larger (e.g., *ctf4*) or smaller (e.g., *cog1*) cell sizes (higher or lower PC1 scores, respectively) or a higher accumulation of cells with delocalized actin patches (e.g., *ypk2*; lower PC2 scores) were defined as the heteroclite mutants, which indicated that a loss-of-function of the 36 corresponding genes (hereafter we refer them as “heteroclite genes”) resulted in morphological changes in specific traits.

### Fitness analysis of the 36 heteroclite mutants

Yeast mutants with an unusual morphology tend to suffer from growth defects. Deletion mutants with an abnormal morphology, not present in the natural yeast strains, may be missing from the natural population due to their slow growth. We examined this possibility in the 36 heteroclite mutants and in the remaining 74 mutants. Fitness was assessed for the nonessential gene deletion strains in rich media [15] to compare the degree of fitness. We found that number of deletion mutants with significantly decreased fitness was similar; 22 (60%) and 39 (58%) gene deletion mutants conferred decreased fitness compared to the wild-type strains in the 36 heteroclite and 74 mutants, respectively. An alignment of these mutants according to their degree of fitness is shown in Additional file 9: Figure S7A. The distribution of the deletion mutants (Additional file 9: Figure S7B) indicated that the fitness of most of the 4718 strains was distributed around a central fitness of 1, whereas the heteroclite and the rest of the gene deletion mutants were distributed at a lower fitness level. No significant differences were observed between the heteroclite mutants and the other mutants (Mann–Whitney *U*-test, *P* =0.72). Thus, many morphological mutants had decreased fitness, but this was not more common in the 36 heteroclite mutants.

**Figure 5:**
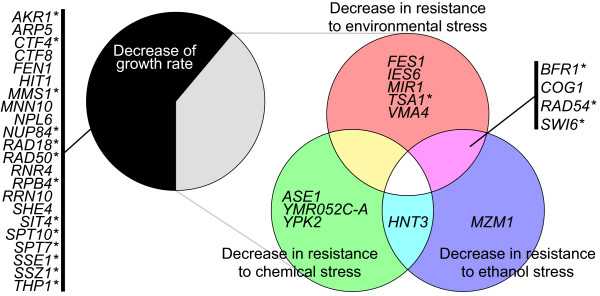
**Summary of the phenotypes annotated to the 36 heteroclite genes.** Annotations of the phenotypes in Additional file 10: Table S3 are summarized. Asterisks indicate the genes annotated to the GOs as detected by MANOVA (Figure 7A and Additional file 12: Table S4).

Of the 36 heteroclite strains, 22 were identified as slow-growth mutants in normal medium [15] (Additional file 10: Table S3 and Figure 5). We found that all of the remaining 14 heteroclite strains were assigned to at least one phenotype relating to a decrease in the stress response, such as “decrease of resistance to environmental stress,” “decrease of resistance to chemical stress,” and “decrease of resistance to ethanol stress” (Additional file 10: Table S3 and Figure 5). This suggested that the loss-of-function mutation of these 14 genes was negatively selected for under these special environmental conditions.

### Protein abundance analysis of the 36 heteroclite genes

Ghaemmaghami et al. [16] carried out genome-wide analyses of the protein levels in *S. cerevisiae*. This data were used to compare the abundance of the protein molecules per cell for the 110 selected genes with deletions resulting in a variable morphology. The protein molecules of the 4718 nonessential genes were distributed across a wide range (from 41.1 − 681,000) as were those of the 110 selected genes (from 149 to 378,000; Additional file 11: Figure S8A). To investigate the differences of the abundance of the protein molecules between the 36 heteroclite genes and the others, a Mann–Whitney *U*-test was performed. We found that no significant differences (Additional file 11: Figure S8B) were detected. This suggested that the morphological abnormalities caused by the deletion of the 36 heteroclite genes were not dependent on protein abundance, and that protein abundance was not a common property in the heteroclite genes.

### Assessment of genetic interactions in the 36 heteroclite genes

**Figure 6:**
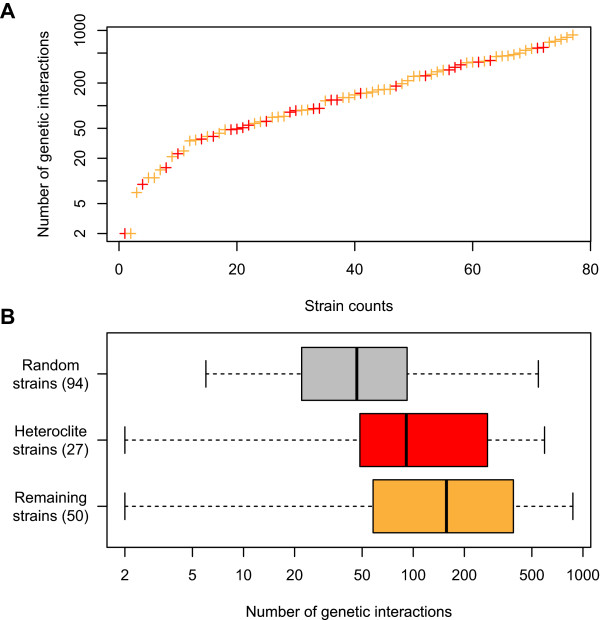
**Distribution of the number of genetic interactions. (A)** Alignment of the gene deletion strains according to the number of genetic interactions. Red and orange crosses indicate the detected strains and the undetected strains, respectively. The number of genetic interactions of 77 strains was counted, as the remainder was not available [17]. **(B)** Boxplot of the number of genetic interactions. Grey, red and orange boxes indicate number of genetic interactions in 100 genes randomly selected from nonessential genes as a reference, heteroclite strains and remaining strains, respectively. Gene number available for genetic interaction data is shown in parentheses.

The degree of genetic interaction correlates with the number of different gene attributes [17]. In this study we investigated the relationship between the morphological extent in the gene deletion mutants and the number of genetic interactions. Stringent cutoff values were imposed to identify the genetic interaction in each gene (*ϵ* <−0.12, *P* <0.05 or *ϵ* >0.16, *P* <0.05). We found that >70% of the selected genes had >50 genetic interactions, with an average number of 180.0 (Figure 6), although about 50% of the randomly selected nonessential genes contained <50 genetic interactions. A Mann–Whitney *U*-test showed this difference to be statistically significant (*P* <0.01). These results suggested that the high morphological diversity of the heteroclite mutants was due to the impact of the genetic interaction.

### Gene annotations frequently observed in the 36 heteroclite genes

To identify the gene ontologies (GOs) that were statistically enriched in the 36 heteroclite genes, a multivariate analysis of variance (MANOVA) was applied. We found that of the 13 GOs that were annotated to at least three of the 36 heteroclite genes (Additional file 12: Table S4, see Methods), nine GOs showed a significant correlation (*P* <0.05 after Bonferroni correction, *F*-test) with PC1, PC2, or PC4, which differentiated the deletion mutants from the natural strains. This implied that these nine GOs were selected due to the morphological phenotype of the deletion mutants.

**Figure 7:**
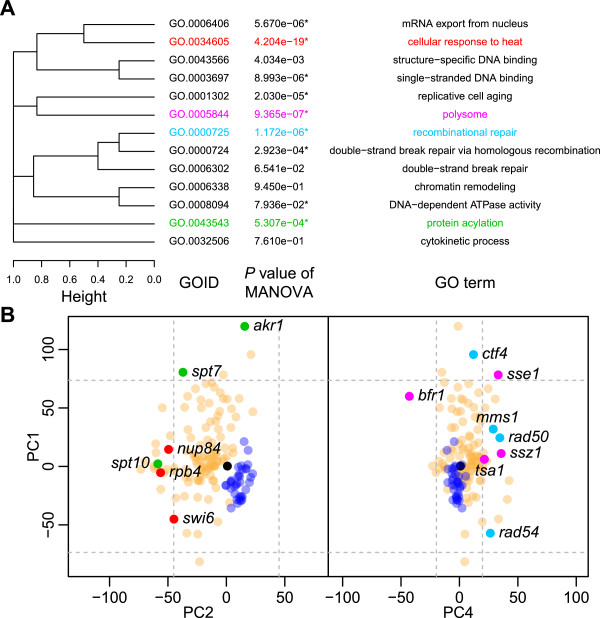
**Annotations of the genes deleted in the heteroclite 36 homozygous mutants. (A)** Hierarchical cluster analysis of the 13 GOs. The dendrogram was constructed with complete linkage with the distances of GOs. The colored GOs indicate the representative four GOs in Figure 7B. The asterisks denote the GOs with significant differences between the annotated genes and the natural strains with PC scores at *P* <0.05 after Bonferroni correction by MANOVA. **(B)** Distribution of PC scores. Blue, orange, and black circles indicate the natural strains, the gene deletion strains, and BY4743, respectively. Red, magenta, cyan, and green circles denote the gene deletion strains with genes annotated to the GO terms of “cellular response to heat” (GO:0034605), “polysome” (GO:00005844), “recombinational repair” (GO:0000725), and “protein acylation” (GO:0043543), respectively. Gray dashed lines indicate the percentile of *P* =0.05 after Bonferroni correction to detect the heteroclite strains by one-sample two-sided tests of normal distribution.

To ascertain the representative GOs, a hierarchical cluster analysis was performed based on the similarity of annotated genes. A dendrogram was constructed based on complete linkage, which highlighted four representative GOs that covered 13 genes (Figure 7A). We found that the GO with the lowest *P-*value (*P* < 5.0 × 10^−19^) was “cellular response to heat” (GO:0034605), which was annotated to *NUP84*, *RPB4*, and *SWI6* (Figure 7B, red). Since this GO, identified by PC2, contributed mostly to the trait, “ratio of the cells with actin localization in bud,” actin localization, was most likely perturbed by the loss-of-function of genes involved in “cellular response to heat.” Likewise, “polysome” (GO:00005844), “recombinational repair” (GO:0000725), and “protein acylation” (GO:0043543) were identified by PC1, PC4, and PC2 (Figure 6A, magenta, cyan, and green circles). Thus, essential GOs and morphological phenotypes conserved in nature were proposed.

## Discussion

The natural yeast strains analyzed were derived from different geographical and ecological origins, and displayed diverse morphological phenotypes [9]. They contained an average of 30,000 single nucleotide polymorphisms (SNPs) and 63 deletion events, with >200 base pairs (bp) per strain [2]. The nonessential gene deletion collection contained a single deletion in every gene, which highlighted the fact that approximately 50% of the deletion mutants had an abnormal morphology compared with their parental strains [6]. Here, we showed a more diverse morphology for the deletion mutants than the natural yeast strains after verifying our procedure with the mosaic progeny and showing the robustness of our analysis for the strain selection. We also identified deletion mutants with morphologies not encountered in the natural yeast population. We propose that these genes are essential gene candidates in nature.

Although natural strains accumulated many nucleotide polymorphisms [2], their morphology was less diverse than the deletion collection, suggesting a robustness of the cell morphology in the natural yeast strains. Several possible explanations exist for the conserved morphology in the natural strains. First, a cellular mechanism may coordinate an increase in cell size with biosynthetic capacity and nutrient availability [18]. Therefore, natural yeast strains likely have an upper size limit. The deviation of the long axis length in the mother cell was 5.3 − 7.1 and 4.5 − 11.4 μm in natural yeast strains and the deletion collection, respectively. These results showed that natural yeast strains were less diverse in cell size. Second, the morphological parameters that directly affect growth rate may be conserved due to the advantages they confer during the competition to survive. When the ratio of unbudded cells increased, an increase in doubling time was expected [19]. The ratio of unbudded cells was between 13.4% and 71.5% in the natural yeast strains [9], implying that the unbudded ratio of 71.5% was constitutive. Third, the loss of cell polarity may result in defects in shmoo formation and in the mating process [20], which could constitute an evolutionary disadvantage. Thus, the evolutionary conservation of morphological traits may link to the survival of the extant budding yeast in nature.

We revealed a higher morphological variation generated by single gene deletions. Yeast deletion mutants with a slow-growth phenotype likely work against natural competition in nature [21]. We found that 22 of the 36 heteroclite deletion mutants had >10% reduction in fitness. In addition to the decreased fitness in the normal medium, the heteroclite deletion mutants were often unable to grow under certain growth conditions, such as high or low temperatures, lack of nutrients, treatment with toxic compounds, and radiation exposure. Survival under severe environment conditions and response to change is essential for preservation of the species [22]. Another possibility is the presence of lesions other than those of vegetative growth. Yeast life cycles are composed of many processes involving different forms and cell shapes [23]. Genes acting on vegetative cellular morphogenesis are sometimes essential for the progression of other yeast life cycles, including germination, mating, meiosis, sporulation, and biofilm formation, which may be confer a competitive advantage in survival. Finally some yeast variants may become disadvantaged during breeding or domestication [24]. Due to their usefulness for alcohol fermentation, *Saccharomyces* yeast species may acquire their competitive advantage under high ethanol conditions.

Some properties were common in the heteroclite genes. The heteroclite genes frequently displayed a relatively large number of genetic interactions because the deletion mutant genes conferred the most variable morphological phenotypes. We hypothesized that the genes with a high number of genetic interactions acted as a hub in the cellular network [17], affecting many aspects of cell morphology. Alternatively, the predominant yeast genes involved in essential cellular processes frequently interact with each other and thus influence cell morphology to a major extent. We also ascertained that the genes belonging to the heat-shock response, DNA repair, translation, and protein modification were enriched in the heteroclite mutants. This led to the speculation that these gene functions are essential in nature; e.g., budding requires genes involved in the heat-shock response. The genes involved in DNA repair are important because this function is required for adaptation or survival under exposure to natural radiation. Since exposure to mild stress leads to an increased tolerance for other stresses [25], these functions may be necessary in preparing for future threats.

## Conclusions

High-dimensional morphometric features of *Saccharomyces cerevisiae* were examined to find that the gene deletion strains had higher morphological variance than the natural strains. Our multivariate analyses revealed gene deletion mutants whose morphologies were not seen in nature. The yeast genes that were practically important in nature were characterized in terms of extent of genetic interaction and specific cellular functions. Although evolution has often been cited in the context of current living species or for those now extinct, this study provided a new insight into the evolution and species conservation of yeast. Further study will be required to fully clarify the evolution of important yeast genes by means of competitive assays with deletion mutants and natural yeast strains.

## Methods

### Strains, culture and morphological analysis

The collection of diploid homozygous deletion strains was purchased from EUROSCARF (the EUROpean Saccharomyces Cerevisiae ARchive for Functional Analysis). The strains were grown in synthetic C medium [12] at 30°C to logarithmic phase.

To obtain the fluorescent images of the cell wall, actin cytoskeleton, and nuclear DNA, yeast cells were stained triply with fluorescein isothiocyanate-ConA (Sigma Aldrich, St. Louis, MO, USA), rhodamine-phalloidin (Invitrogen, Carlsbad, CA, USA), and 4′,6-diamidino-2-phenylindole (Sigma Aldrich), respectively. At least 200 cells were counted on the fluorescent images from one independent culture, and we visualized sets of images from each homozygous diploid strain. The image sets were processed with the CalMorph software (version 1.3, designed for diploid cells) as described previously [12]. Raw images and datasets are freely available at http://www.yeast.ib.k.u-tokyo.ac.jp/natural_vs_deletion/index.html. All statistical analyses were performed using R software (http://www.r-project.org). To investigate the distribution of the morphological changes, a PCA was performed based on the variance–covariance matrix of the *Z*-score using the prcomp() function in R.

### Statistical models to estimate the effects on cell morphology

To statistically assess the morphological differences of the cells, the GLM, an extension of the normal linear model, was used, which applied not only a Gaussian but also other probability distributions. The models of the probability distributions for the 501 parameters were determined to accommodate the statistical model used in the GLM.

Of the 501 parameters calculated by CalMorph, 220 parameters were coefficients of variation (CV) of their related mean parameters calculated from a single cell trait. The CV parameters depended highly on the mean trait values, and this dependence could be uncoupled by a nonlinear Lowess regression method [7]. To normalize the CV values by uncoupling the dependency, the Lowess regression of the CV values by the mean values was performed using the lowess() function of R with a smooth span of 0.4, as described previously [9]. CVs were assumed to be Gaussian-distributed after the normalization. A further 183 parameters, representing the mean cell morphologies with positive continuous values, were assumed to be gamma-distributed. Another 37 parameters, representing the mean cell morphologies with continuous values ranging from zero to one, were assumed to be beta-distributed. The remaining 61 parameters, representing the ratio of cells in specimen, were assumed to be binomially distributed with overdispersion. The models of the probability distributions and descriptions of the parameters are listed in Additional file 13: Table S5. The single linear model was used for the assessment of the Gaussian, gamma, and beta parameters in the GLM as defined by

1

where *η* is link function listed in Additional file 13: Table S5 , *y*_*i*_ is the response variable (parameter values), *β*_0_ is the intercept, *β*_1_ is the slope (fixed effect), *x*_*i*_ is the explanatory variable (0 and 1 for the wild-type and the mutants, respectively), and *ϵ*_*i*_ is the error. For the BY, RM and their segregants, a single value for each culture was estimated in each of 501 parameters after three replications. For the 36 natural strains and BY4743, a single value for each strain was estimated in each parameter after five and 40 replications, respectively, with three of the 501 parameters being discarded due to a missing value. The Wald test for the maximum likelihood estimation of *β*_1_ was used as the *Z*-score in this study.

### Selection of representative diploid gene deletion strains by Mahalanobis distance

To select the homozygous diploid gene deletion strains for the morphological analysis, we calculated the Mahalanobis distance of each of 4718 haploid from a central distribution of wild-type strains. *Z*-values of 501 parameters were calculated by assuming one of four probability distribution models for each parameter (Additional file 13: Table S5) and were used to calculate the Mahalanobis distance using the mahalanobis() function in R. We selected 100 strains with the highest Mahalanobis distance scores (Additional file 2: Figure S2A) and 20 strains located at the edge of the PC1-PC20 space of 4718 gene deletion strains (Additional file 2: Figure S2B), making a total of 120 strains (Additional file 3: Table S1) covering 7.4% of the total 4718 haploid strains distance. These 120 strains were investigated; the diploid morphology of 110 strains was successfully analyzed, with 10 strains failing to supply any morphological data due to poor growth (Additional file 3: Table S1 and Additional file 2: Figure S2C). In addition, we analyzed the morphology of the next most variable 20 strains with Mahalanobis distance scores (Additional file 3: Table S1) to confirm the robustness of the analyses.

### Estimation of phenotypic variance and detection of gene deletion strains beyond the variation of wild yeasts

To estimate the variance between BY and RM as shown in Figure 2B, we used the published data [12]. We calculated the sum of squares of the *Z*-value after it was centered by the mean values of BY (n =3) and RM (n =3), respectively, and divided by 4 (the degrees of freedom). For the 37 natural strains in Figure 3A, we used the published data [9]. The sum of squares was calculated for each parameter after the Z-values were centered by the mean of the 37 strains, and divided by 36 (the degrees of freedom) not to be affected by the sample size. For the 110 gene deletion strains, the *Z*-values were centered by the mean of 110 strains, and the sum of the squares was divided by 109. To estimate the equiprobability density ellipses in two-dimensional space as shown in Figures 2D and 3C, the variance–covariance matrices were calculated from the PC scores after centering, as described above, and the probability density of the multivariate normal distribution was estimated for each of 200 × 200 bins on the two-dimensional space of each pair of PCs using the dmvnorm() function in R. The equiprobability density ellipse was drawn using the contour() function in R. To detect the gene deletion strains beyond the variance of the natural strains, the variance of the 37 natural strains was estimated by centering with the mean of BY4743, and a one-sample two-sided test of normal distribution from the mean of BY4743 was applied for each gene deletion strain in each PC. The Mann–Whitney U-test with corrections for both of continuity and ties was performed using wilcox.test() function in R.

### Multivariate analysis of variance (MANOVA) to detected gene ontologies (GOs) annotated to genes conferring morphological variation from the natural strains

To detect the GOs annotated to genes in which deletions caused morphological differences between the natural strains and the homozygous gene deletion strains, 13 GOs were selected that were annotated to at least three of the 36 genes among all the GOs annotated to <100 genes in a genome. Out of the 36 genes, 21 were annotated to at least one of the 13 GOs (Additional file 6: Table S2 and Additional file 12: Table S4). We performed a single linear model as below


where the *Y* phenotypes consist of three PCs (PC1, PC2, and PC4) as a factor of the background strains, and natural and homozygous gene deletion strains with a BY background. χ was a factor of the type of mutation defined by GO, where the wild type and natural strains had no artificial gene deletion (0 as a dummy variable) but where the homozygous gene deletion strains did have a gene deletion (1 as a dummy variable). Then we applied the linear model function (lm() function in R) to the null hypothesis: PC1 + PC2 + PC4 = *s* (cell morphology was explained only by the background of each strain) and the alternative hypothesis: PC1 + PC2 + PC4 = *s* + *x* (cell morphology was explained by background and mutation type). These two hypotheses were assessed by *F*-test. Finally, each of the 13 GOs (Additional file 12: Table S4) was subjected to a MANOVA with the natural strains and the wild-type BY4743, and the gene deletion strains were annotated to each of the 13 GOs.

## Electronic supplementary material

Additional file 1: Figure S1: Pair plots of PC scores for BY, RM, and segregants. Blue, green, and gray circles indicate BY, RM, and segregants, respectively. Red and black ellipses show equiprobability density ellipses. (PDF 220 KB)

Additional file 2: Figure S2: Distribution of the Mahalanobis distance of haploid gene deletion strains. (A) Histogram of the Mahalanobis distances of 4718 nonessential gene deletion mutants. Gray and yellow boxes indicate the frequency of 4718 mutants and 122 replicated wild-type (*his3*) strains. The red arrow indicates the 100^th^ mutant. (B) Distribution of PC1 and PC2 scores of 4718 mutants. Red and gray circles denote the top 100 mutants and the remainder, respectively. The green circle represents an outlying mutant. (C) Alignment of the gene deletion strains according to the Mahalanobis distance. Red, black and green crosses indicate the mutants with higher Mahalanobis distance, the mutants discounted because of growth defects, the 20 mutants selected to distribute outside of the PC coverage, respectively. (D) Boxplot of Mahalanobis distance. Mahalanobis distance from a center of distribution of wild-type strain (BY4743) was calculated from the Z-scores. (PDF 5 MB)

Additional file 3: Table S1: Mahalanobis distance of the gene deletion mutants. The 110 mutants used in this study were selected by Mahalanobis distance or the principal components listed in the table. (XLSX 18 KB)

Additional file 4: Figure S3: Pair plots of PC scores for the natural strains and the gene deletion strains. Blue, orange, and black circles indicate the natural strains, the gene deletion strain, and BY4743, respectively. Blue and orange ellipses denote the equiprobability density ellipses of the natural strains and the gene deletion strains. Ratio of variance was calculated by gene-deletion/natural. (PDF 695 KB)

Additional file 5: Figure S4: Distribution of the cumulative contribution ratio of the PCA on the *Z*-values of the natural strains and the gene deletion strains. Black bars indicate the contribution ratio of each PC (left axis). The red curve shows the cumulative contribution ratio (right axis). Red circles denote the cumulative contribution ratio of the first four PCs at 60% of the cumulative contribution ratio. (PDF 105 KB)

Additional file 6: Table S2: *P* values of the one-sample test for the identification of the deletion mutants having an abnormal morphology not seen in the natural yeast strains. Asterisk indicates significant *P* value at *P* <0.05 with the Bonferroni correction (n =440). (XLSX 20 KB)

Additional file 7: Figure S5: Summary of the 36 heteroclite gene deletion strains. (A) Distribution of the number of genes deleted in the heteroclite strains as detected in each of the PCs. (B) Distribution of the number of genes deleted in the heteroclite strains detected in each PC. (PDF 428 KB)

Additional file 8: Figure S6: PC loadings and independent morphological features. The 1^st^ PCA indicates the PCA for the *Z*-values of the natural strains and the 110 gene deletion strains. The 2^nd^ PCA indicates the PCA for the *Z*-values of 40 replicates of BY4743 as null-distributed data. The PCs in the 1^st^ PCA column indicate significant loadings between the indicated PCs and the parameters. The parameters among same PCs in 2^nd^ PCA column are likely to be correlated with each other, and these parameter traits should be interpreted as being biologically the same. (XLSX 26 KB)

Additional file 9: Figure S7: Distribution of fitness. (A) Alignment of the gene deletion strains according to fitness [15]. Red and orange crosses indicate the heteroclite and the remaining genes, respectively. Of the 110 strains, fitness data were available for 103 [15]. (B) Distribution of fitness of 4711 mutants of the nonessential genes. Gray boxes in the upper panel show a histogram of fitness for the 4711 mutants. Gene number available for fitness data is shown in parentheses [15]. (PDF 402 KB)

Additional file 10: Table S3: The phenotypes annotated to the 36 heteroclite genes. Annotations were collected from the phenotypes listed in the *Saccharomyces* genome database (http://www.yeastgenome.org/). (XLSX 14 KB)

Additional file 11: Figure S8: Distribution of protein abundance. (A) Alignment of gene deletion strains according to the protein abundance [16]. Red and orange crosses indicate the heteroclite and the remaining genes, respectively. Of the 110 strains, 83 strains were available for protein abundance data [16]. (B) Distribution of abundance for 3868 proteins. Gray boxes at the upper panel indicate the histogram of abundance for the 3868 proteins. Gene number available for protein abundance data is shown in parentheses [16]. (PDF 409 KB)

Additional file 12: Table S4: List of the GOs annotated to at least three of the 36 heteroclite genes. *P* values were calculated by the MANOVA. (XLSX 11 KB)

Additional file 13: Table S5: The models of the probability distributions and descriptions of the 501 parameters. (XLSX 28 KB)
