# Supplementary figures and images for: Unveiling nonessential gene deletions that confer significant morphological phenotypes beyond natural yeast strains
Source: BMC Genomics. 2014 Oct 25;15(1):932. doi: 10.1186/1471-2164-15-932 (PMC4221665; doi:10.1186/1471-2164-15-932)

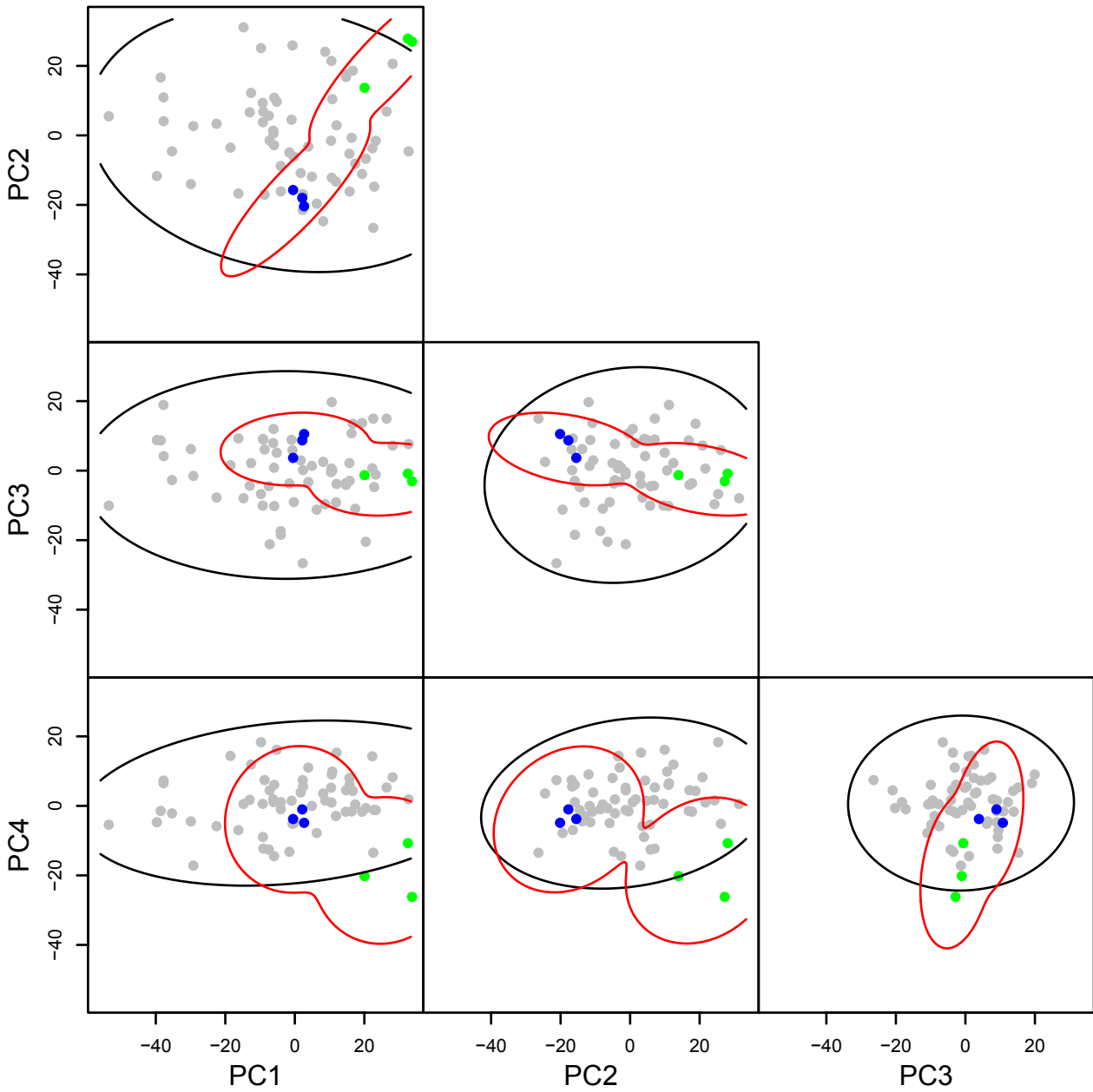

Supplement: Supplementary file 1 — Additional file 1: Figure S1: Pair plots of PC scores for BY, RM, and segregants. Blue, green, and gray circles indicate BY, RM, and segregants, respectively. Red and black ellipses show equiprobability density ellipses. (PDF 220 KB) [file 12864_2014_6623_MOESM1_ESM.pdf]

**A**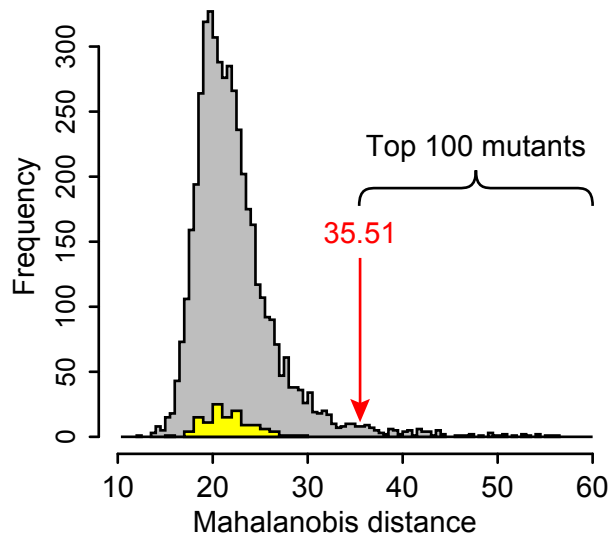**B**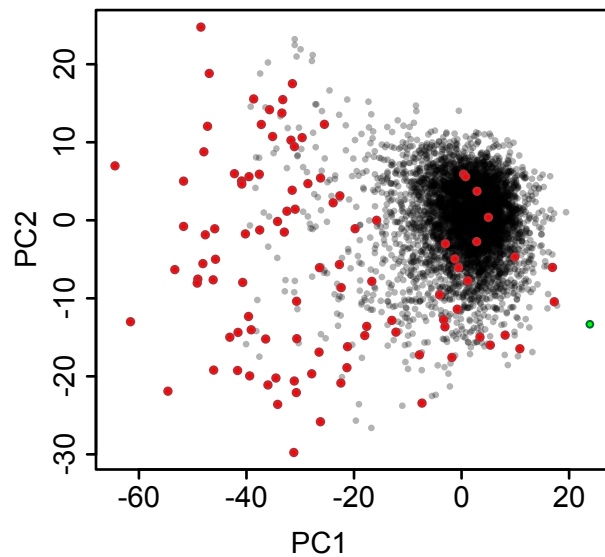**C**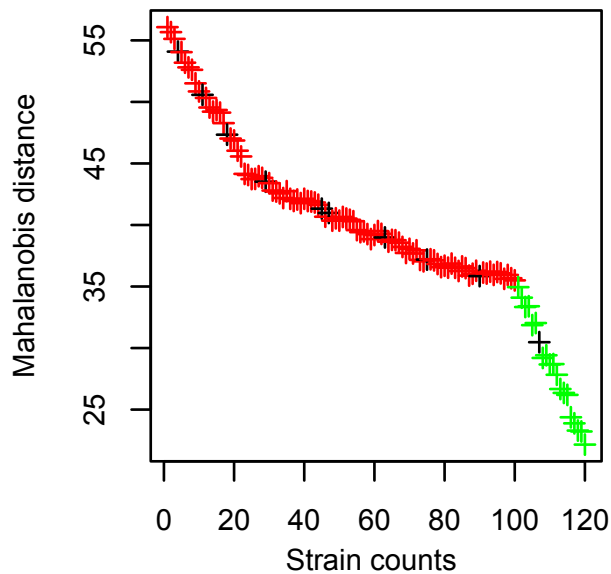**D**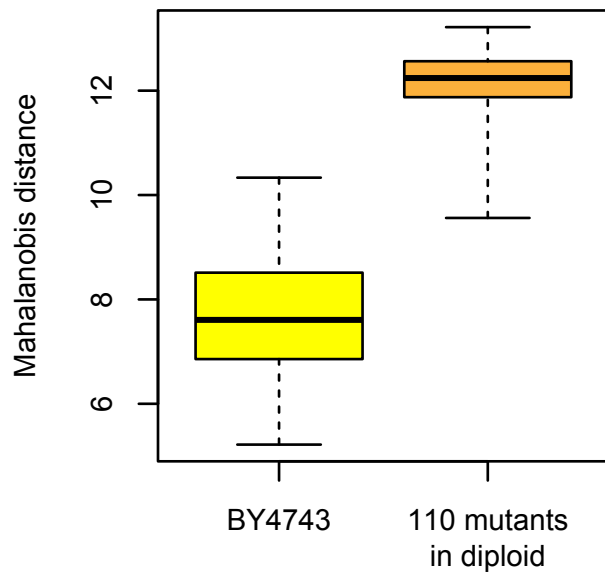

Supplement: Supplementary file 2 — Additional file 2: Figure S2: Distribution of the Mahalanobis distance of haploid gene deletion strains. (A) Histogram of the Mahalanobis distances of 4718 nonessential gene deletion mutants. Gray and yellow boxes indicate the frequency of 4718 mutants and 122 replicated wild-type (his3) strains. The red arrow indicates the 100th mutant. (B) Distribution of PC1 and PC2 scores of 4718 mutants. Red and gray circles denote the top 100 mutants and the remainder, respectively. The green circle represents an outlying mutant. (C) Alignment of the gene deletion strains according to the Mahalanobis distance. Red, black and green crosses indicate the mutants with higher Mahalanobis distance, the mutants discounted because of growth defects, the 20 mutants selected to distribute outside of the PC coverage, respectively. (D) Boxplot of Mahalanobis distance. Mahalanobis distance from a center of distribution of wild-type strain (BY4743) was calculated from the Z-scores. (PDF 5 MB) [file 12864_2014_6623_MOESM2_ESM.pdf]

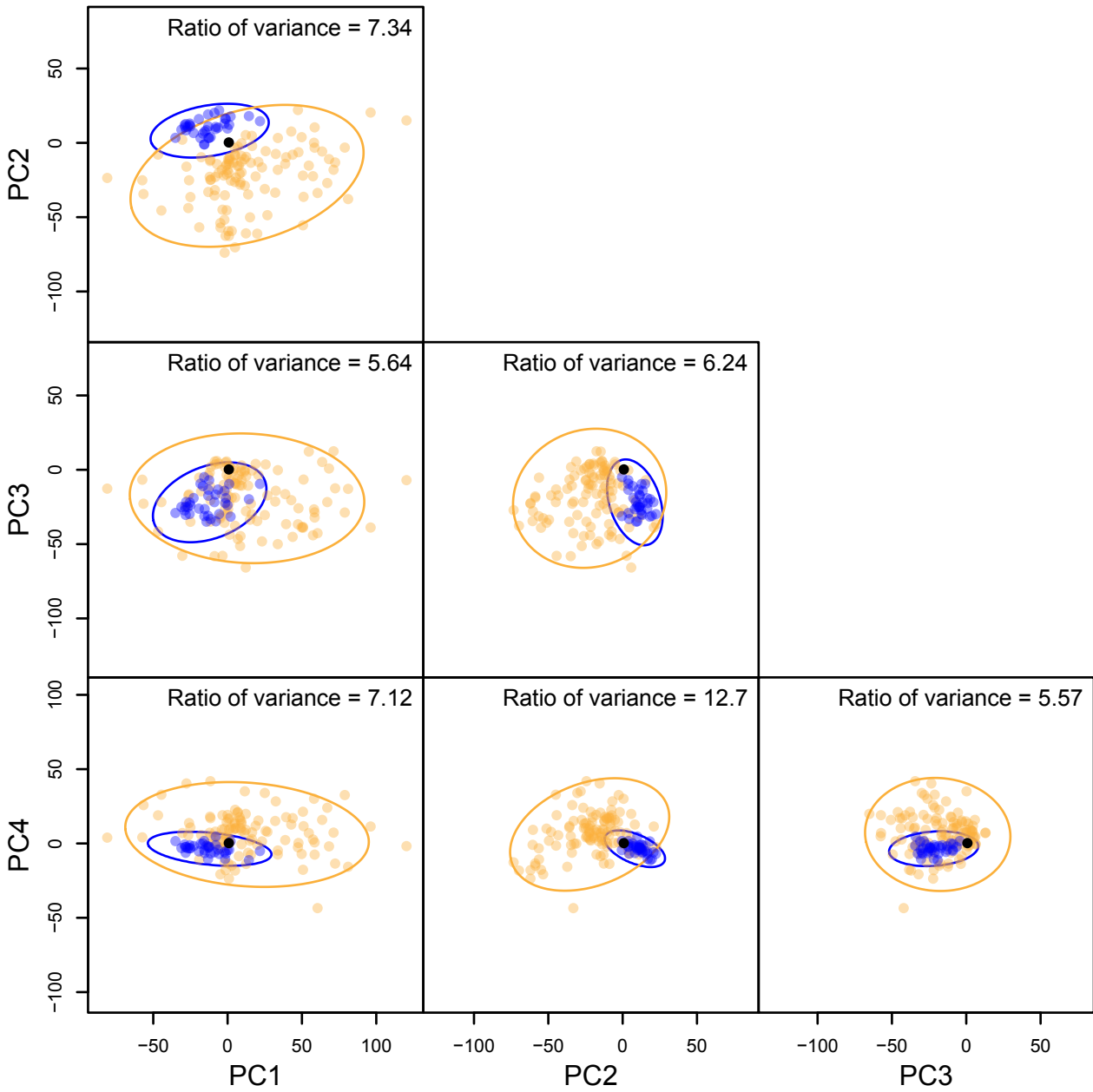

Supplement: Supplementary file 4 — Additional file 4: Figure S3: Pair plots of PC scores for the natural strains and the gene deletion strains. Blue, orange, and black circles indicate the natural strains, the gene deletion strain, and BY4743, respectively. Blue and orange ellipses denote the equiprobability density ellipses of the natural strains and the gene deletion strains. Ratio of variance was calculated by gene-deletion/natural. (PDF 695 KB) [file 12864_2014_6623_MOESM4_ESM.pdf]

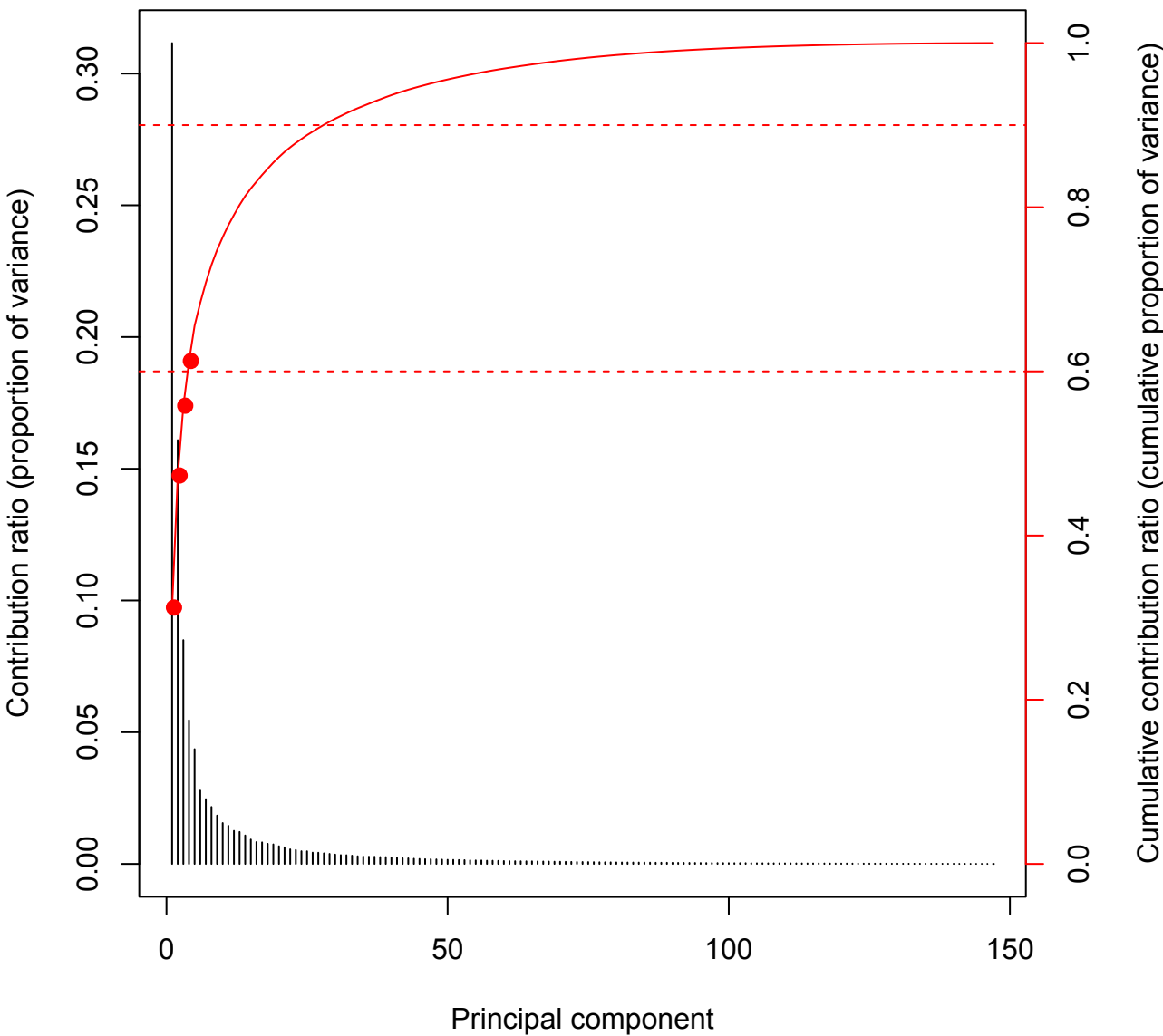

Supplement: Supplementary file 5 — Additional file 5: Figure S4: Distribution of the cumulative contribution ratio of the PCA on the Z-values of the natural strains and the gene deletion strains. Black bars indicate the contribution ratio of each PC (left axis). The red curve shows the cumulative contribution ratio (right axis). Red circles denote the cumulative contribution ratio of the first four PCs at 60% of the cumulative contribution ratio. (PDF 105 KB) [file 12864_2014_6623_MOESM5_ESM.pdf]

**A**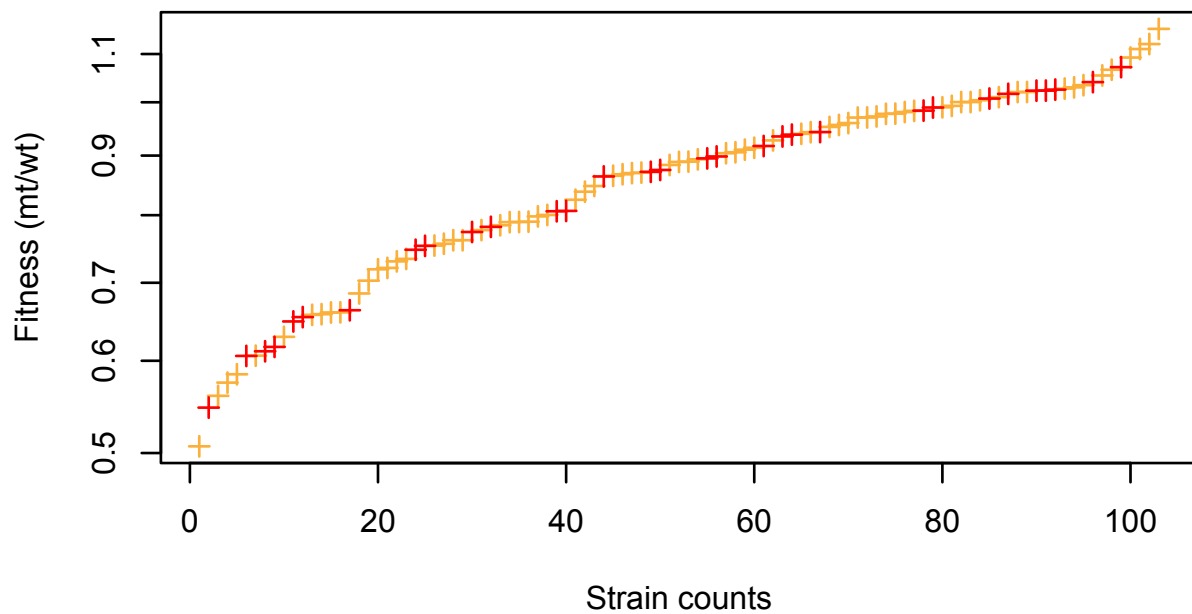**B**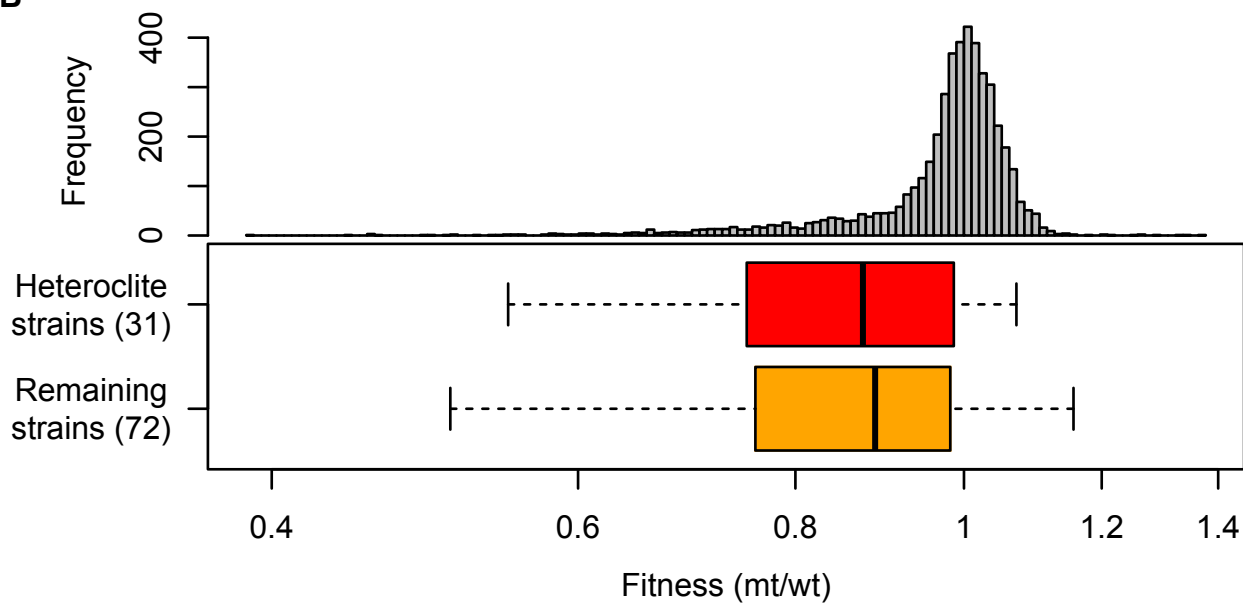

Supplement: Supplementary file 9 — Additional file 9: Figure S7: Distribution of fitness. (A) Alignment of the gene deletion strains according to fitness [15]. Red and orange crosses indicate the heteroclite and the remaining genes, respectively. Of the 110 strains, fitness data were available for 103 [15]. (B) Distribution of fitness of 4711 mutants of the nonessential genes. Gray boxes in the upper panel show a histogram of fitness for the 4711 mutants. Gene number available for fitness data is shown in parentheses [15]. (PDF 402 KB) [file 12864_2014_6623_MOESM9_ESM.pdf]

**A**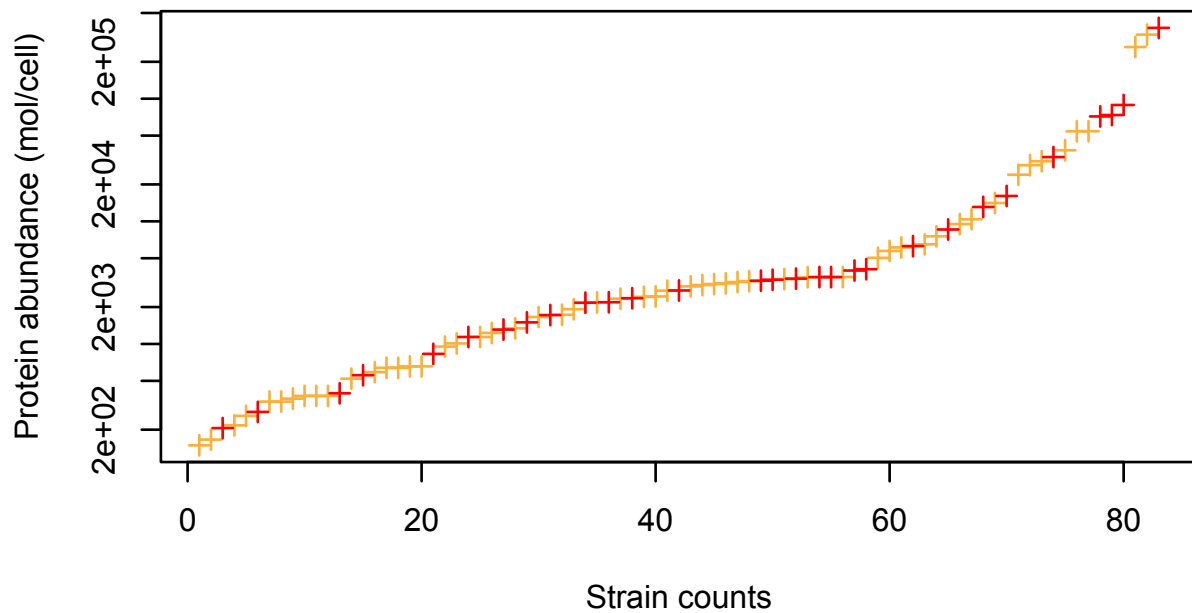**B**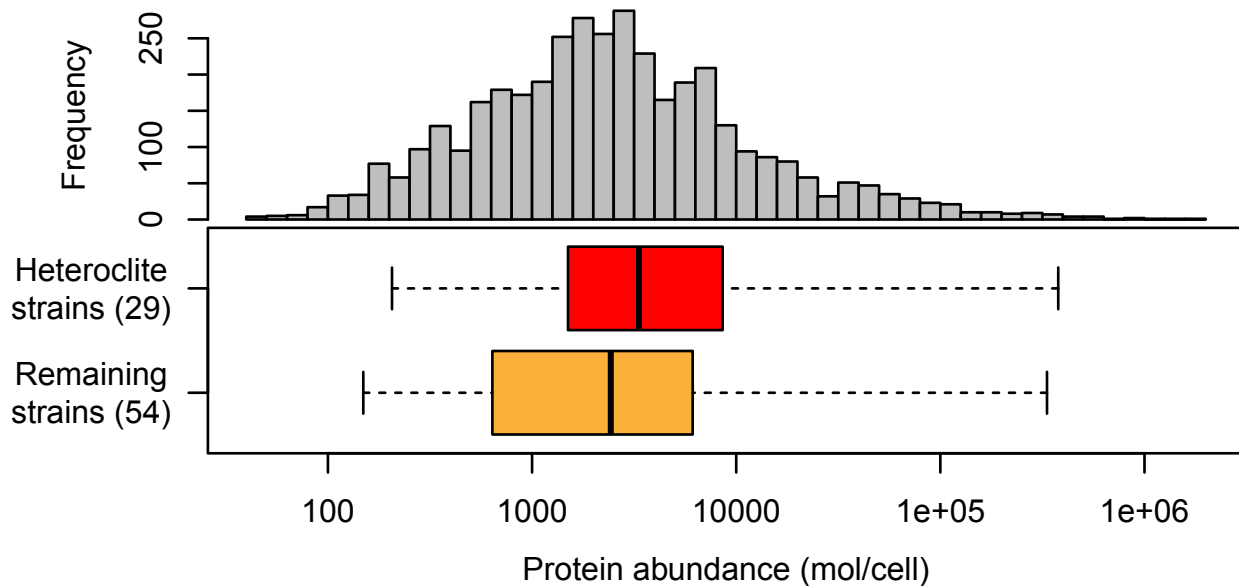

Supplement: Supplementary file 11 — Additional file 11: Figure S8: Distribution of protein abundance. (A) Alignment of gene deletion strains according to the protein abundance [16]. Red and orange crosses indicate the heteroclite and the remaining genes, respectively. Of the 110 strains, 83 strains were available for protein abundance data [16]. (B) Distribution of abundance for 3868 proteins. Gray boxes at the upper panel indicate the histogram of abundance for the 3868 proteins. Gene number available for protein abundance data is shown in parentheses [16]. (PDF 409 KB) [file 12864_2014_6623_MOESM11_ESM.pdf]
