# Supplementary material for: Unveiling nonessential gene deletions that confer significant morphological phenotypes beyond natural yeast strains
Source: BMC Genomics. 2014 Oct 25;15(1):932. doi: 10.1186/1471-2164-15-932 (PMC4221665; doi:10.1186/1471-2164-15-932)
Supplement: Supplementary file 7 — Additional file 7: Figure S5: Summary of the 36 heteroclite gene deletion strains. (A) Distribution of the number of genes deleted in the heteroclite strains as detected in each of the PCs. (B) Distribution of the number of genes deleted in the heteroclite strains detected in each PC. (PDF 428 KB) [file 12864_2014_6623_MOESM7_ESM.pdf]

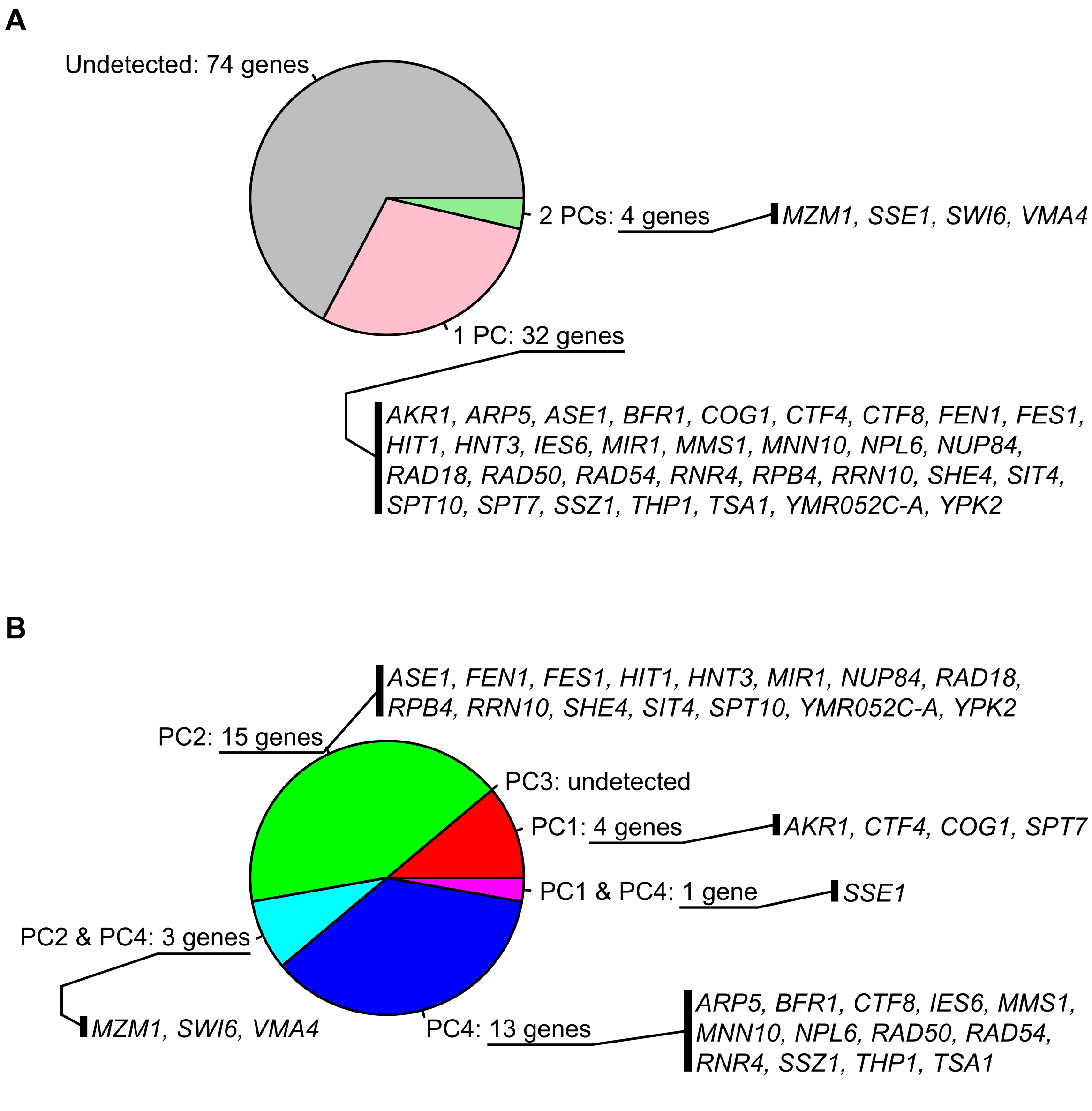
A
Undetected: 74 genes
2 PCs: 4 genes
MZM1, SSE1, SWI6, VMA4
1 PC: 32 genes
AKR1, ARP5, ASE1, BFR1, COG1, CTF4, CTF8, FEN1, FES1, HIT1, HNT3, IES6, MIR1, MMS1, MNN10, NPL6, NUP84, RAD18, RAD50, RAD54, RNR4, RPB4, RRN10, SHE4, SIT4, SPT10, SPT7, SSZ1, THP1, TSA1, YMR052C-A, YPK2
B
ASE1, FEN1, FES1, HIT1, HNT3, MIR1, NUP84, RAD18, RPB4, RRN10, SHE4, SIT4, SPT10, YMR052C-A, YPK2
PC2: 15 genes
PC3: undetected
PC1: 4 genes
AKR1, CTF4, COG1, SPT7
PC1 & PC4: 1 gene
SSE1
PC2 & PC4: 3 genes
MZM1, SWI6, VMA4
PC4: 13 genes
ARP5, BFR1, CTF8, IES6, MMS1, MNN10, NPL6, RAD50, RAD54, RNR4, SSZ1, THP1, TSA1
